# Time to see the bigger picture: Individual differences in the attentional blink

**DOI:** 10.3758/s13423-015-0977-2

**Published:** 2015-11-17

**Authors:** Charlotte Willems, Sander Martens

**Affiliations:** 1 Department of Neuroscience, University Medical Center Groningen, Groningen, The Netherlands; 2 Neuroimaging Center, University of Groningen, P.O. Box 196, 9700 AD Groningen, The Netherlands

**Keywords:** Attentional blink, Individual differences, Temporal selective attention, Visual perception

## Abstract

If two to-be-identified targets are presented in close temporal succession, identification for the second target is typically impaired. This attentional blink (AB) phenomenon has long been considered as a robust, universal cognitive limitation. However, more recent studies have demonstrated that AB task performance greatly differs between individuals, with some individuals even showing no AB in certain paradigms. Several studies have focused on these individual differences in an attempt to reveal the mechanism underlying the AB, but an overview of this approach is currently missing. Here, by reviewing studies regarding individual differences in AB task performance, we investigate how individual differences have contributed to our understanding of the AB. We show that the individual differences AB literature provides reliable indications that the AB is a multifaceted phenomenon that presumably arises from a combination of factors; individuals with higher levels of executive working memory (WM) functioning and broad attentional focus perform better in the AB paradigm than individuals with lower executive functioning of WM and narrow attentional focus. As it turns out, seeing the bigger picture certainly seems helpful for AB task performance.

**Electronic supplementary material:**

The online version of this article (doi:10.3758/s13423-015-0977-2) contains supplementary material, which is available to authorized users.

## The attentional blink: An individual differences approach

**Fig. 1:**
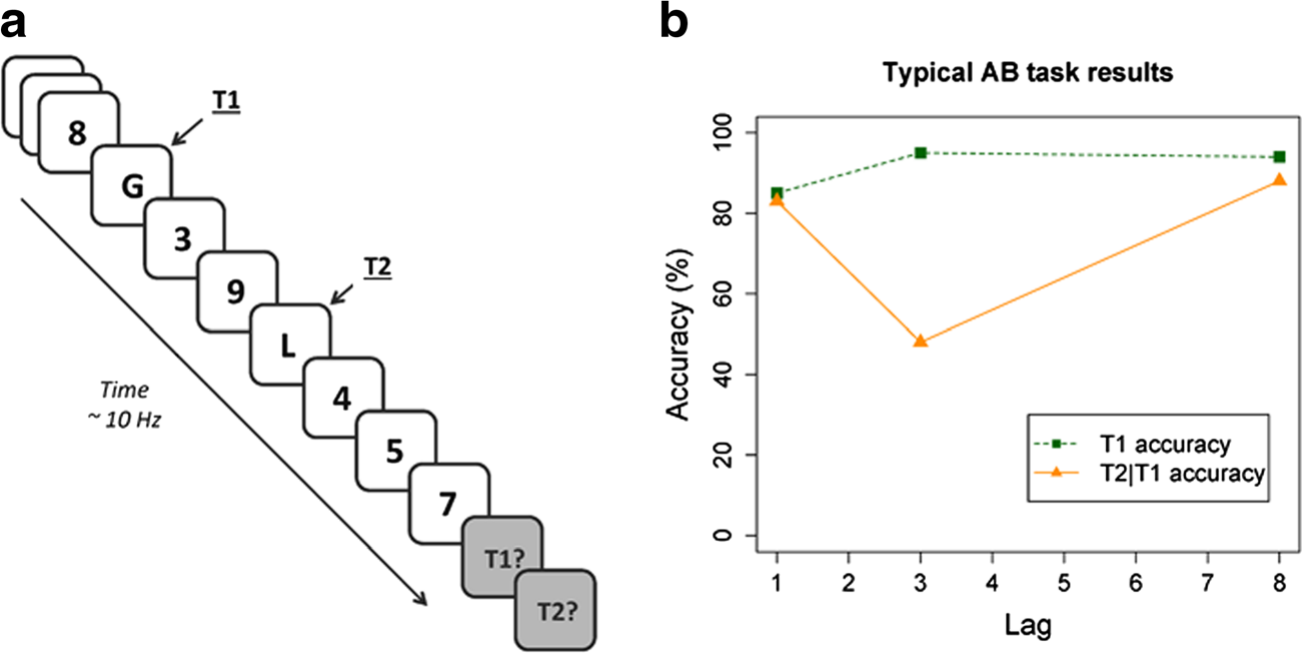
**a**) The design and **b**) the results of a typical AB task

Every waking moment, we are surrounded by an overload of visual information that is nowadays only increasing as a result of modern technology. To deal with this information, selective attention plays a crucial role in assuring that attention is allocated to relevant information instead of irrelevant information, e.g., to a traffic sign instead of a commercial billboard. This system works well when one piece of information, i.e., a single target, has to be identified. However, temporal selective attention starts to fail when a second to-be-identified target is presented in close temporal succession of the first target. This cognitive limitation is called the Attentional Blink (AB; Raymond et al., 1992), and its origin can be systematically studied with the AB paradigm, revealing the cognitive processes that underlie selection and consolidation of information in the temporal dimension. Here, as depicted in Fig. 1a, two target stimuli embedded in a Rapid Serial Visual Presentation (RSVP) stream of distractor stimuli (~10 Hz) have to be identified, and reported after the stream ends. Typically, as shown in Fig. 1b, first target (T1) accuracy is close to ceiling, but when the second target (T2) follows the first one in close temporal proximity (200–500 ms), the rate of accurate T2 reports drops drastically. In case no intervening distractors are presented between the two targets, or the lag between T1 and T2 increases, T2 accuracy approaches T1 accuracy (for reviews see: Dux & Marois, 2009; MacLean & Arnell, 2012; Martens & Wyble, 2010). By virtue of the fact that the AB occurs on some trials, but not on others with identical sensory input, both failures and successes of temporal selective attention, working memory (WM), and conscious awareness can be compared.

The source of the AB has been widely debated over the last twenty years. Whereas earlier studies have focused on a role of resource depletion (e.g., Chun & Potter, 1995), more recently, evidence has been revealed that the AB may at least partly result from adverse attentional strategies (e.g., Di Lollo, Kawahara, Shahab Ghorashi, & Enns, 2005; Nieuwenstein, Chun, Van der Lubbe, & Hooge, 2005; Olivers & Nieuwenhuis, 2005, 2006; Taatgen, Juvina, Schipper, Borst, & Martens, 2009; Wierda, van Rijn, Taatgen, & Martens, 2010; Wyble, Bowman, & Nieuwenstein, 2009). That is, although there is evidence for a role of capacity limitations of short-term WM (Chun & Potter, 1995; Dell’Acqua et al., 2012; Duncan, Ward, & Shapiro, 1994), it has been shown that AB task performance can be enhanced through either manipulation (Arend et al., 2006; Ferlazzo et al., 2007; Nieuwenstein & Potter, 2006; Olivers & Nieuwenhuis, 2006; Taatgen et al., 2009; Wierda et al., 2010) or training (Choi et al., 2012; Oei & Patterson, 2013; Reedijk, Bolders, Colzato, & Hommel, 2015; Tang et al., 2013; Willems, Damsma, Wierda, Taatgen, & Martens, 2015). This suggests that changing attentional strategies can alter AB task performance, perhaps comprising faster processing or the relocation of attentional resources.

One approach to further investigate the nature of the AB is to study individual differences. Although the AB phenomenon has long been considered to be a fundamental, universal limitation, large individual differences exist in AB task performance (e.g., Dale & Arnell, 2010; Feinstein, Stein, Castillo, & Paulus, 2004; Martens, Munneke, Smid, & Johnson, 2006b; McLaughlin, Shore, & Klein, 2001). Under certain task conditions, there are even individuals—sometimes referred to as non-blinkers—who show little or no AB (e.g., Feinstein et al., 2004; Martens, Munneke, et al., 2006; Troche & Rammsayer, 2013). Studying the variability of AB magnitudes throughout the population can help to construct a more complete and detailed picture of the dynamics of temporal selective attention. To this end, in the last fifteen years, a substantial body of research has focused on individual differences in AB magnitude, disclosing important clues regarding the nature of the AB. However, in spite of multiple reviews written about the AB phenomenon in general (Dux & Marois, 2009; Hommel et al., 2006; MacLean & Arnell, 2012; Martens & Wyble, 2010), an overview of studies regarding individual differences is currently missing in the literature. Therefore, by providing such an overview, here, we will reveal the state of the art in the individual differences AB literature. First, we will address the reliability of individual AB task performance within an AB task, between different AB tasks and related tasks, and as a function of time. Second, we aim to reveal the origin of individual AB task performance, where we will focus on indications regarding the adverse attentional strategy that is said to underlie the AB.

## Methods

Two different databases with peer-reviewed literature, i.e., PubMed and PsycINFO, were searched with the search strings as presented in Table 1. The last search was performed on August 18th, 2015, and papers were included according to the following criteria: 1) The study concerns the AB paradigm as used to measure the dynamics of temporal selective attention. 2) The study concerns healthy participants. 3) The study investigates differences between individuals regarding AB task performance, or investigates the relationship between individual AB task performance and other factors. This resulted in the inclusion of 68 papers, marked with a “*” in the reference list and summarized in Table 2, included as supplementary information (SI).

**Table 1:** The search strings as used to search the selected databases

*Database*	*Search string*
PsycINFO	(DE "Attentional Blink" OR TX ( attention* AND (blink* OR nonblink*) ) ) AND (DE "Individual Differences" OR TX ( individual* OR develop* OR magnitud* ) )
PubMed	("Attentional Blink"[Mesh] OR (attention*[tw] AND (blink*[tw] OR nonblink*[tw]))) AND ("Individuality"[Mesh] OR individual*[tw] OR development*[tw] OR magnitud*[tw])

Throughout the selected papers, different statistical techniques have been employed to analyze the data, which is indicated per study in Table 2 (see SI). Although a detailed discussion of the statistical approach of these studies lies outside the scope of this review, it should be noted that the splitting of continuous data into (extreme) groups and possible additional dichotomization is accompanied with certain costs, including inflated effect sizes and p-values (MacCallum, Zhang, Preacher, & Rucker, 2002; Preacher, Rucker, MacCallum, & Nicewander, 2005). Although such studies can certainly be meaningful, these results should be interpreted with caution. Furthermore, these results need to be replicated in future studies employing a continuous approach of the data. That is, the disadvantages of splitting continuous data are in potential averted when analyzing the sample as a continuum whenever possible, where the use of generalized linear mixed models is preferred over repeated measures analyses of variance (Baayen, Davidson, & Bates, 2008; Bolker et al., 2009).

## Reliability of individual AB task performance

### Individual AB magnitude within tasks

Although methods have been proposed that can either attenuate (Arend et al., 2006; Oei & Patterson, 2013; Olivers & Nieuwenhuis, 2005, 2006; Taatgen et al., 2009; Wierda et al., 2010), or resolve the AB (Choi et al., 2012; Reedijk et al., 2015), it is generally found that individual AB magnitude cannot be reduced by simply practicing the task (Braun, 1998). Evidence for this was also provided in Dale and Arnell (2013), and Dale, Dux, and Arnell (2013), where the internal-consistency reliability was tested within different variations of the AB paradigm that are common in the literature. Using a split-half procedure, performance within tasks was found to correlate reasonably high; Spearman-Brown corrected *r* ranged from .48 to .91, and .54 to .76 in Dale and Arnell (2013), and Dale et al. (2013), respectively. Further evidence for the reliability of individual performance within tasks was inter alia revealed in Martens and Johnson (2009), and Martens and Valchev (2009), where Spearman-Brown prophecy coefficients were > .84 for AB magnitude, > .83 for T1 accuracy, and > .91 for T2 accuracy given correct report of T1, i.e., T2|T1.

### Individual AB magnitude across tasks

Individual AB task performance has also been found to be reliable between tasks. To the best of our knowledge, the first evidence that individual AB task performance is stable between an AB task and an AB-like task was reported by McLaughlin et al. (2001). They found a positive relationship between individual AB task performance and performance on a so-called attentional dwell time task (Duncan et al., 1994), in which participants had to identify two masked targets with varying lags between the two targets, i.e., lacking the typical distractor stimuli of the AB paradigm. Note however that individual AB magnitude has been found to differ dependent on task conditions such as stimulus category and duration, or the modality in which the RSVP is presented (Heinz et al., 2007; Martens, Dun, Wyble, & Potter, 2010; Martens, Johnson, Bolle, & Borst, 2009; Martens, Wierda, Dun, de Vries, & Smid, 2015; Martens, Kandula, & Duncan, 2010; Martens, Korucuoglu, Smid, & Nieuwenstein, 2010; Willems, Wierda, Viegen, & Martens, 2013). For example, it was found that individuals who showed no AB when target selection could be based on alphanumerical information did show a drop in T2 accuracy when the RSVP contained picture stimuli, T1 was rotated, or when targets had to be identified based on color (Martens, Dun, et al., 2010; Martens, Korucuoglu, et al., 2010; Willems et al., 2013). Despite these findings, intra-individual differences between AB tasks as used throughout the literature are assumed to be stable; Dale et al. (2013), and Kelly and Dux (2011) showed reasonably high correlations between intra-individual performance in AB paradigms in which target selection had to be based on either category or feature information (*r* > .43). Furthermore, they compared AB tasks containing similar instructions for T1 and T2 detection with AB tasks containing a task-switch between T1 and T2 detection, e.g., “Identify the letter in a stream of digits (T1), and determine whether this letter was followed by a white X (T2)”. Relations between performance on a task with task-switch and performance on a task without task-switch were found to be reliable (*r* > .21) (Dale & Arnell, 2013; Dale et al., 2013, but see: Kelly & Dux, 2011, who failed to find such a relationship), although intra-individual performances on two tasks without task-switch were stronger related. Dale and colleagues concluded that in spite of shared variability in the task-switch vs. no task-switch comparison, inclusion of a task-switch does introduce variability that is unrelated to the AB.

### Individual AB magnitude over time

Performance thus seems to be fairly stable within the timespan of one experimental session, but what about a longer time span? Dale and Arnell (2013), and Dale et al. (2013) reported that individual AB task performance was stable over a time period of 7–10 days (*r* > .39). However, throughout the course of life, changes can be observed in the pattern of individual AB task performance. Because the temporal selective attention system is still developing during childhood, children under the age of 10 do not yet show the typical hook-shaped pattern as seen in adults (Garrad-Cole, Shapiro, & Thierry, 2011; Heim, Benasich, Wirth, & Keil, 2013; Heim, Wirth, & Keil, 2011). Instead, young children show the largest dip in performance at lag 1, after which T2 accuracy recovers linearly. Furthermore, the slope of this recovery is smaller than commonly seen in adults. Around the age of 10–11, the more typical AB pattern emerges in the performance of children, and from this point up to adulthood, a general increase in performance is observed (Garrad-Cole et al., 2011; Heim et al., 2013; 2011).

Around the age of forty, individual AB task performance is thought to reach its peak, after which a trend of decline sets in (Georgiou-Karistianis et al., 2007). As measured in adults over the age of 60, the AB of older individuals is more pronounced and lasts longer in time (Georgiou-Karistianis et al., 2007; Jain & Kar, 2014; Lahar, Isaak, & McArthur, 2001; Maciokas & Crognale, 2003; Male, Sheppard, & Bradshaw, 2009; Shih, 2009; van Leeuwen, Müller, & Melloni, 2009). In addition, overall single-target accuracy has been found to be lower compared to younger adults (Jain & Kar, 2014; Maciokas & Crognale, 2003). According to the inhibitory deficit hypothesis (Hasher & Zacks, 1988), this decline in performance is the result of the decreased ability to inhibit irrelevant information when growing older. In the AB paradigm, this inability to ignore distracting stimuli would cause problems in target selection, which is in line with studies marking the inability to suppress distractor stimuli as a source of the AB, as will be discussed below (Dux & Marois, 2008; Martens & Valchev, 2009; Olivers & Watson, 2006). Taken together, these studies suggest that age differences can partly explain individual differences in AB magnitude, particularly in children and older adults. In a sample of young to middle-aged adults, age differences are less likely to play a role, and individual AB task performance is therefore considered to be stable over time.

## Deployment of attentional control

### The role of working memory

Assuming that individual differences in the AB are stable within tasks, across tasks, and over time, studying the origin of these differences can reliably inform us about the nature of the AB. To this end, studies have focused on the relation between AB magnitude and individual differences in WM, given its key role in target selection and identification. In this context, it is important to note that WM functioning is assumed to consist of storage capacity on the one hand, and executive functioning on the other hand (Baddeley, 1996).

In order to examine the role of executive WM in relation to AB magnitude, Colzato, Spapé, Pannebakker, and Hommel (2007) measured individual performance in the operation span (OSPAN) paradigm, which measures the ability of participants to remember words while internal repetition is prevented by an additional mathematics task. They revealed a negative relationship between WM operational span and AB magnitude. Moreover, this relation held after they controlled for the level of fluid intelligence, often associated with individual WM functioning. This finding was replicated by Arnell, Stokes, MacLean, and Gicante (2010), where a higher OSPAN score resulted in a smaller AB magnitude when they controlled for fluid intelligence, reading comprehension and rate, and digit span. Because these latter measures are thought to represent the static storage capacity of WM, it was hypothesized that AB task performance is likely to be influenced by the level of executive functioning of WM, but not by storage capacity of WM (Arnell et al., 2010). In line with this, Arnell and Stubitz (2010) showed that individual AB magnitude can be predicted by filtering efficiency of WM, but not by visual WM storage capacity. These results do not only confirm the role of executive WM functioning, but also suggest that the individual ability to keep irrelevant information out of WM is important for individual AB task performance. Martens and Johnson (2009), though, did not find a relation between individual AB magnitude and executive WM, measured by symmetry span and reading span. They also found no evidence for a relation between AB magnitude and short-term memory measures, thought to represent storage capacity, or between AB magnitude and fluid intelligence. Taken together, these studies consistently suggest that both storage capacity of WM, and fluid intelligence are unrelated to individual AB task performance (Arnell et al., 2010; Arnell & Stubitz, 2010; Colzato et al., 2007; Klein, Arend, Beauducel, & Shapiro, 2011; Martens & Johnson, 2009; Troche, Indermühle, & Rammsayer, 2012; Wagner, Rammsayer, Schweizer, & Troche, 2014). However, the operational component of WM can be seen as modulator of AB magnitude, such that individuals who exhibit higher levels of executive functioning show smaller AB magnitudes (Arnell et al., 2010; Arnell & Stubitz, 2010; Colzato et al., 2007, but see: Martens & Johnson, 2009). In line with this, non-blinkers have been found to update representations in WM at a faster rate than blinkers (Martens, Munneke, et al., 2006; Troche & Rammsayer, 2013). This was indicated by the findings of earlier latencies of the P3 component in EEG analyses, irrespective of target position or lag. Thus, these results show that the AB is not likely to be the result of a structural bottleneck in static capacity limitations, but that operational capacities of WM regarding management of incoming information are important for individual AB task performance.

These results are further confirmed by studies revealing a relation between AB magnitude and the neurotransmitter striatal dopamine (DA), which can be considered to be a key player in WM functioning. However, the direction of this relationship remains unclear. Slagter et al. (2012), who measured striatal dopamine using PET scans, showed that higher levels of striatal dopamine D2-like receptor binding, i.e., lower levels of endogenous dopamine, were related to larger AB magnitudes. In line with this, Colzato, Slagter, Spapé, and Hommel (2008) found a negative relationship between spontaneous Eye Blink Rate (sEBR)—a marker of central dopaminergic functioning—and individual AB size, such that individuals with low basal dopaminergic activity showed a larger AB. A note of criticism here may be that the latter result was based on a correlation analysis in a small sample, and should therefore be considered with caution. Especially because Slagter and Georgopoulou (2013) failed to replicate the relationship between sEBR and AB magnitude.

In contrast, concerning genetic predisposition related to the efficacy of dopaminergic neurotransmission, Colzato, Slagter, De Rover, and Hommel (2011) showed a relation between individual AB magnitude and the dopamine receptor D2 (DRD2) C957T polymorphism. This polymorphism is associated with striatal DA/D2, and was tested because the DA/D2 nigrostriatal pathway has been found to be important for executive WM (Cools, Gibbs, Miyakawa, Jagust, & D’Esposito, 2008). Colzato and colleagues showed that DRD2 C957T T/T-carriers, who are assumed to have lower levels of striatal DA/D2, displayed a smaller AB than C-allele carriers. Furthermore, AB task performance could not be related to polymorphisms associated with frontal dopamine, thought to be involved in static maintenance of information. However, Felten et al. (2013) failed to replicate the relationship between AB magnitude and the DRD2 C957T polymorphism, in spite of their large sample and attempts to rule out additional confounding factors. A final example of the complexity of this topic is illustrated by Reedijk et al. (2015), who showed that presentation of alpha-frequency binaural beats can resolve the AB, but only in individuals with low sEBR, i.e., low striatal dopamine.

An explanation for these conflicting results has been proposed by Slagter et al. (2012). Following Cools and D’Esposito (2011), they hypothesized that the relationship between the level of striatal dopamine and AB magnitude may actually be u-shaped, where either too little or too much dopamine would hurt AB task performance. However, Slagter et al. presented no evidence to support this claim. So, despite indications that dopamine, as representative of WM functioning, plays a role in accounting for individual AB task performance, the precise nature of this relationship remains a topic for future research.

### Inhibition of irrelevant information

One way in which higher-level executive WM could benefit AB task performance is by efficient inhibition of distracting information. Indeed, as mentioned before, Arnell and Stubitz (2010) found a relation between AB magnitude and WM filtering efficiency, and the deeper and longer-lasting AB of older adults was attributed to the deteriorated ability to inhibit irrelevant information (Georgiou-Karistianis et al., 2007; Jain & Kar, 2014; Lahar et al., 2001; Maciokas & Crognale, 2003; Male et al., 2009; Shih, 2009; van Leeuwen et al., 2009).

The importance of the ability to ignore irrelevant information was also suggested by Dux and Marois (2008), who showed that sensitivity to a priming cue could predict individual AB magnitude. That is, by priming the identity of T2 with a cue presented in the RSVP, they found that the size of the AB predicted how much performance improved as a result of priming, such that large blinkers showed the largest decrease in AB magnitude when T2 was primed. This suggests that target-irrelevant information in the RSVP is better inhibited in small blinkers than in large blinkers (but see: Slagter & Georgopoulou, 2013, who suggest that the length rather than the depth of the AB can be predicted by sensitivity to priming).

In line with this, Martens and Valchev (2009) compared an attentional dwell time task containing only two targets and two masking distractors with a regular AB task, i.e., an RSVP with two masked targets embedded in distractor stimuli. They showed that whereas task performance of blinkers suffered from the extra distracting stimuli in the RSVP, performance of non-blinkers was not influenced by this manipulation. Moreover, using EEG, it was found that non-blinkers showed less distractor-related frontal activity in trials where no targets appeared than blinkers (Martens, Munneke, et al., 2006), suggesting that non-blinkers pay less attention to distractors in the RSVP than blinkers do.

A personality characteristic that is associated with efficient inhibition of irrelevant information and limitations for sustained attention is impulsiveness (Dickman, 2000). In adolescents, it was found that higher levels of impulsiveness were related to a deeper, and more protracted AB compared to lower levels of impulsiveness (Li, Chen, Lin, & Yang, 2005). Subsequently, Troche and Rammsayer (2013) made a distinction between dysfunctional impulsiveness, i.e., the tendency to act without forethought in a situation where this is disadvantageous, and functional impulsiveness, i.e., the tendency to act without forethought in a situation where this is beneficial. They found that non-blinkers scored higher on functional impulsivity, associated with higher speed of processing and more efficient processing (Dickman, 2000), but no difference was found regarding dysfunctional impulsivity. These results seem to be incompatible with those reported by Li et al. (2005), because the measuring scale used by Li et al. is thought to measure mainly dysfunctional impulsivity. Further research is therefore needed to clarify the relationship between individual AB task performance and both the level of dysfunctional impulsivity and the level of functional impulsivity.

Not directly in line with the assumption that inhibition of distractors is beneficial for AB performance is the finding that bilingual individuals, claimed to exhibit enhanced inhibitory control, showed a more pronounced AB than monolingual individuals (Colzato, Bajo, et al., 2008; Khare, Verma, Kar, Srinivasan, & Brysbaert, 2013). However, in other cognitive tasks, Colzato, Bajo et al. (2008) found no differences in active inhibitory efficiency between bilinguals and monolinguals. Therefore, they argued that bilinguals might be better in selecting goal-relevant information when this is competing with goal-irrelevant information, because of their habit to keep two languages separate. Thus, rather than a difference in inhibitory control for the suppression of distractors, it seems that bilinguals invest more attention in processing goal-relevant information, i.e., target selection, which results in an enhanced AB in bilinguals when compared to monolinguals.

Interestingly, the suggested improved ability to ignore distractor stimuli for small blinkers does not seem to be linked to increased control over attentional capture, i.e., when a salient distractor impairs the visual search for a unique target. That is, Kawahara and Kihara (2011) did not find evidence for a relationship between AB magnitude and sensitivity to attentional capture. However, mixed results have been found with regard to habitual video game players, who are argued to exert improved control over exogenous attentional capture based on their heightened experience with visual distraction during video gaming (Cain, Prinzmetal, Shimamura, & Landau, 2014). Whereas one study showed that experienced video gamers have smaller AB magnitudes than non-video gamers (Green & Bavelier, 2003), this could not be replicated in another study (Cain et al., 2014). Furthermore, it has been shown that AB task performance can be trained by playing action video games, although not by other types of video gaming (Green & Bavelier, 2003; Oei & Patterson, 2013). Next to improved control over attentional capture, though, Oei and Patterson (2013) proposed that the enhanced AB task performance might as well be the result of improved switching of attention between items, because this is a frequently needed skill in action video gaming. Therefore, the effect of frequent video gaming on AB task performance and the role of individual differences awaits further investigation, as does the role of attentional capture.

Finally, it must be noted that the importance of distractor inhibition in the AB paradigm may be influenced by discriminability of targets among distractor stimuli. That is, Willems et al. (2013) showed that neither small blinkers nor large blinkers showed much suppression of distractor stimuli when target selection had to be based on color instead of alphanumerical information. This was confirmed by findings of Bourassa, Vachon, and Brisson (2015), who performed an EEG study with a similar letter-only RSVP. They showed that in case of an erroneous T2 report at lag 3, a P3 was detected for the distractor letter following T2. Furthermore, they showed that individuals with lower lag-3 accuracy, showed higher P3 amplitudes, and thus, responded stronger to the distractor following T2 than individuals with higher accuracy. Thus, in a paradigm with low discriminability, Bourassa et al. also found no evidence for suppressed distractors, but for delayed attentional selection. In addition, Visser and Ohan (2012) revealed that participants who are faster information processors—as indicated by a rapid automatized naming task—have an advantage in the AB paradigm when the RSVP contained highly similar targets and distractor stimuli. However, faster processing was not found to be a predictive factor if targets and distractors were easier to distinguish. Therefore, the importance of inhibition of distractor stimuli may depend on the level of difficulty to discriminate distractors from targets, whereas processing speed may be more relevant when this distinction becomes more difficult.

### Speed of processing

Processing speed alone, though, does not appear to be a strong determining factor for AB magnitude. However, it can be seen as predictor for the level of overall target accuracy. For example, in a sample of 8–10 year olds, overall mean T2|T1 performance was linked to normal developing reading ability (McLean, Stuart, Visser, & Castles, 2009). But whereas both general reading ability and mean T2|T1 accuracy were related to speed of processing, the level of reading ability and processing speed were not related to AB magnitude. Moreover, Arnell, Howe, Joanisse, and Klein (2006) revealed that AB magnitude could not be predicted by cognitive non-RSVP measures that require comparable information-processing abilities as the AB task, including tasks that require speeded responses. However, reaction time regarding speeded manual and vocal identification of single stimuli was related to general target accuracy in the RSVP. Therefore, speed of information processing is thought to be predictive for target accuracy, but not for individual AB magnitude per se.

### Too much attention can hurt performance

Given that attentional control may help to efficiently select targets, and to ignore irrelevant information, one would expect that higher attentional investment in target identification would be beneficial for AB task performance. Paradoxically, though, it was found that adding an extra task next to the RSVP task caused performance to improve. Olivers and Nieuwenhuis (2005; 2006) showed that listening to music or thinking about holiday plans during the RSVP presentation resulted in a decreased AB magnitude (but see Footnote 1 in Olivers & Nieuwenhuis, 2006, where it is noted that attempts to replicate the latter result failed or showed a substantially smaller effect). Furthermore, Wierda et al. (2010) and Taatgen et al. (2009) found that discriminating the presence of a red dot during the AB task resulted in a smaller AB magnitude. It appears that broadening of attention that is allocated to the RSVP results in better AB task performance. However, the hypothesis that loosening cognitive control by adding an extra task is beneficial for AB task performance seems to contradict with the findings that higher inhibition of distractors leads to better task performance. But where the earlier discussed inhibition of distracting information regards task-relevant distracting information in the RSVP, the distracting tasks as presented in Olivers and Nieuwenhuis (2005; 2006), Wierda et al. (2010), Taatgen et al. (2009), as well as in Arend et al. (2006) are all additional, RSVP-irrelevant tasks.

One explanation for the beneficial effect of an extra task might be that this task enforces a more shallow level of stimulus processing. More specifically, it has been suggested that participants may have a suboptimal processing strategy in which too much attention is allocated to the first target and subsequent distractors, lowering chances of successful report of T2 (Olivers & Nieuwenhuis, 2005, 2006; Shapiro, Schmitz, Martens, Hommel, & Schnitzler, 2006; Taatgen et al., 2009; Wierda et al., 2010). This overinvestment hypothesis is supported by a number of studies showing that attentional investment to T1 is higher on trials where T2 was identified incorrectly, i.e., blink trials, compared to trials where T2 was identified correctly, i.e., no-blink trials (Maclean & Arnell, 2011; Martens, Munneke, et al., 2006; Slagter et al., 2010; Wierda, van Rijn, Taatgen, & Martens, 2012).

By using magnetoencephalography, Shapiro et al. (2006) also revealed that higher attentional investment to T1 resulted in larger AB magnitudes, though it should be noted that this correlation was based on a sample of N = 10. Furthermore, Wu and Hillman (2013) found that children with higher levels of physical fitness perform better in the AB paradigm than lower fit children, in line with other studies that indicate a positive relation between psychical activity, cognitive performance, and brain health (Hillman, Erickson, & Kramer, 2008). As indicated by EEG analyses of the P3 component, it was found that higher fit children invest less attention in T1 processing during the AB period, and less attention to T2 throughout the task. Wu and Hillman argued that these results may be due to higher control over the distribution of attentional resources in case of higher aerobic fitness. In contrast, though, other studies only found a weak relationship between individual P3 amplitudes and T2 identification rate (Martens, Elmallah, London, & Johnson, 2006; McArthur, Budd, & Michie, 1999; Wagner, Rammsayer, Schweizer, & Troche, 2015). These studies show that the relation between P3 amplitude as indicator of attentional investment and individual AB task performance is definitely in need of further research (see for example Wagner et al., 2015, for ideas on future research regarding this relationship).

In support of the idea that control over attentional investment is related to AB magnitude, Dale and Arnell (2010; 2014) showed that dispositional attentional focus is related to individual AB task performance, discriminating between either a diffused attentional processing style or a focused attentional processing style. They tested individuals with the global–local task, where a large stimulus is constructed from a set of smaller stimuli, i.e., the global level and local level, respectively. These levels can either be congruent or incongruent (Navon, 1977). By using multiple variants of this global–local task, Dale and Arnell (2010, 2014) revealed that on the one hand, precedence towards a more diffused attentional style correlated negatively with the size of the AB. On the other hand, precedence towards a more focused attentional style correlated positively with AB magnitude. Moreover, it was found that large blinkers invest more in performance monitoring, which is associated with modulation of cognitive control (MacLean & Arnell, 2013). Here, large AB magnitudes were related to large electrophysiological reactions to performance feedback, indicating high investment in outcome of performance and cognitive control.

In line with this, Thomson, Ralph, Besner, and Smilek (2014) revealed that individuals who were more frequently engaged in mind wandering showed smaller AB magnitudes, as measured with subjective reports. Interestingly, in daily life tasks (e.g., driving, reading), as well as laboratory tasks (e.g., flanker task), mind wandering has been reported as detrimental for performance (Smallwood, McSpadden, & Schooler, 2008; Thomson et al., 2014). Mind wandering, assumed to result in the failure to inhibit task-irrelevant thoughts, has therefore been suggested to consume attentional resources necessary for task execution (McVay & Kane, 2011; Smallwood, 2013). This confirms the idea that—in the context of the AB—mind wandering can reduce attentional control such that it promotes a more broadly distributed rather than focused allocation of attention, and thus, enhances AB task performance.

Perhaps somewhat related to mind wandering, others found that attentional engagement during rest, i.e., when individuals are not engaged in a goal-directed task, occurred more strongly in small blinkers than in large blinkers (MacLean, Arnell, & Cote, 2012). By measuring oscillatory activity, MacLean et al. showed that activity within the alpha and beta frequency bands during resting state was predictive for the size of the AB. Whereas higher alpha activity was associated with larger AB magnitudes, higher beta-band activity was related to smaller AB magnitudes. In addition, individuals with relatively more beta- than alpha-band activity displayed a smaller AB than individuals where the ratio of alpha and beta activity was the other way around. Because alpha waves in waking state are thought to be a sign of an unoccupied cortex, MacLean et al. suggested a negative association between attentional engagement during rest and the size of the AB.

The finding that non-religious individuals displayed a smaller AB magnitude than religious people, here defined as neo-Calvinists, was also attributed to a difference in cognitive processing style (Colzato, Hommel, & Shapiro, 2010). Because Calvinism is based on a pillar concept of society where everyone minds their own business, Calvinists are thought to have a more narrow, focused processing style compared to atheists, who thus were assumed to have a broader, more diffused processing style (Colzato et al., 2010). Thus, a more open attentional processing style due to choice of religion seems to be profitable for individual AB task performance. These findings are in need of replication, however.

MacLean, Arnell, and Busseri (2010) showed that individual AB task performance is also modulated by dispositional affect, where positive dispositional affect is associated with diffused attention, and negative affect with focused attention. Measured with the Positive and Negative Affect Schedule, it was found that on the one hand, positive dispositional affect was predictive for a smaller AB magnitude, whereas on the other hand, negative affect was related to a larger AB magnitude. In addition, MacLean and Arnell (2010) showed that personality traits that are thought to be related to either positive affect or negative affect can modulate individual AB task performance. That is, greater extraversion can be seen as indicative for positive affect, and was negatively related to AB magnitude, whereas greater neuroticism—associated with negative affect—was positively related to AB magnitude. MacLean and Arnell (2010) also argued that openness to experience would result in smaller AB magnitudes, but Kranczioch and Thorne (2013) did not find any evidence for this relationship. Taken together, these studies suggest that dispositional affect and personality traits, as associated with attentional focus, can be seen as modulators for AB magnitude.

Finally, comparison of different meditation styles also showed the beneficial influence of broad over narrow attentional focus. In a sample of experienced meditators, Van Vugt and Slagter (2014) compared meditation where attention is focused on one point, such as an object or thought, with open monitoring (OM) meditation, which means that thoughts can come in and let go during the meditation session. They found that for very experienced meditators (mean = 10,704 hrs) the OM style was beneficial over the focused attention style when applied during the AB task. In addition, Slagter et al. (2007) showed that after an intensive training of OM meditation, participants performed better on the AB task compared to a control group. Here, individuals who showed the largest decrease in attention allocated to T1, as indicated by the T1-elicited P3b, also showed the largest improvement of AB task performance (Slagter et al., 2007). Furthermore, this decrease in attention to T1 was found to relate to a decrease of phase variability in the theta frequency band, indicating that individuals with the largest improvement in AB task performance following the meditation training were ready earlier in time to react to new target information (Slagter, Lutz, Greischar, Nieuwenhuis, & Davidson, 2009).

In line with this, Van Leeuwen et al. (2009) revealed that the age-related decline in AB task performance as seen in older adults seems to be limited if individuals acquire a substantial level of meditation throughout life. Moreover, Braboszcz et al. (2013) also found a reduction of AB magnitude as a result of meditation, testing participants before and after a three-month retreat of Isha-yoga practice, a combination of focused meditation and open monitoring. However, in contrast with these results that reveal a beneficial effect of OM meditation, Braboszcz et al. (2013) found that previous meditation experience with Shoonya yoga, a practice that can be explained either as open or as focused meditation, correlated negatively with AB task performance, such that more advanced meditators showed larger AB magnitudes. However, this latter result may be due to the difficulty of obtaining a strict separation between focused and OM meditation in experienced meditators, especially because all participants had experience with additional forms of meditation practices. Nevertheless, it can be tentatively concluded that practice of OM meditation, promoting an open attentional focus, has a beneficial effect on individual AB task performance.

## Discussion

In summary, individual differences in the AB paradigm have proven to be a reliable source of information regarding the nature of the AB. Furthermore, the individual differences AB literature provides indications that the AB is a multifaceted phenomenon that presumably arises from a combination of factors. First, the literature reveals that the executive component of WM can be seen as a modulator in the process of selection and consolidation of targets, where individuals with a higher operational span exhibit smaller AB magnitudes. These results are at least partly confirmed at the neurophysiological level by findings regarding the neurotransmitter striatal dopamine, serving as representative of WM functioning. These latter findings remain in need of further research, however. Furthermore, the timing and/or the rate of WM updating seem to be relevant, where earlier WM updating is related to better AB task performance. One way in which higher executive functioning seems to benefit AB task performance is in the ability to keep irrelevant information out of WM, i.e., to inhibit distracting information as presented in the RSVP.

Second, the literature suggests that individual AB task performance is determined by the distribution of attention during an AB task. On the one hand, a narrow focus of attention seems to lead to attentional overinvestment to T1 identification, which subsequently causes T2 to be missed when it succeeds T1 in close temporal proximity. On the other hand, a broad focus of attention seems to provide more optimal circumstances under which both targets can be identified when these are presented in a short time frame. This focus of attention has been linked to factors as dispositional affect, personality traits, and lifestyle.

How executive WM functioning and the span of attentional focus are interlinked with regard to the AB awaits further investigation. In relation to the neural correlates of the AB, it would be particularly interesting to examine the suggestion of Slagter et al. (2012) that the relation between dopamine and AB magnitude might be U-shaped. Furthermore, with regard to these future studies, it would certainly benefit the field of individual AB differences to acknowledge and critically discuss the strengths and weaknesses of different statistical techniques applied throughout the literature.

In conclusion, the individual differences AB literature has contributed much to understanding the workings of the temporal selective attention system in the AB paradigm; individuals with higher levels of executive WM functioning, and broad attentional focus perform better in the AB paradigm than individuals with lower executive functioning of WM, and narrow attentional focus. As it turns out, seeing the bigger picture certainly seems helpful for AB task performance.

## Electronic supplementary material

Below is the link to the electronic supplementary material.

ESM 1 (PDF 138 kb)
