# Supplementary material for: Time to see the bigger picture: Individual differences in the attentional blink
Source: Psychon Bull Rev. 2015 Nov 17;23(5):1289–99. doi: 10.3758/s13423-015-0977-2 (PMC5050248; doi:10.3758/s13423-015-0977-2)
Supplement: Supplementary file 1 — (PDF 138 kb) [file 13423_2015_977_MOESM1_ESM.pdf]

Supplementary information

Table 2. Summary of all papers that have been included for the review regarding individual differences in the AB.

Title and authors (year)	N*	Task-switch	T1/T2	Distractor stimuli	Physiological measure	Extra task**	Statistical method***
Relationships between attentional blink magnitude, RSVP target accuracy, and performance on other cognitive tasks, Arnell, Howe, Joanisse & Klein (2006)	64	No	Specific RAN elements: color, digit, letter or object picture	Similar stimuli as the targets	x	RAN (rapid automatized naming) task; Manual RT task; Delayed RT task; Vocal naming task; Location probe task	regression/correlation
Executive control processes of working memory predict attentional blink magnitude over and above storage capacity, Arnell, Stokes, MacLean & Gicante (2010)	50	Yes	T1: Red letter; word; object picture, T2: Specific letter; word; object picture	Black letters; words; object pictures	x	Nelson-Danny reading test; Forward digit span; Backward digit span; OSPAN; Raven's Standard Progressive Matrices (SPM)	RM-ANOVA/correlation
Attentional blink magnitude is predicted by the ability to keep irrelevant material out of working memory, Arnell & Stubitz (2010)	60	Yes	T1: White letter, T2: Black X	Black letters	x	Filtering efficiency task; WM capacity task	RM-ANOVA/RM-ANCOVA
Failure of temporal selectivity: Electrophysiological evidence for (mis)selection of distractors during the attentional blink, Bourassa, Vachon & Brisson (2015)	24	No	Red letters	Black leters	EEG	x	RM-ANOVA/correlation
Plasticity of visual attention in Isha yoga meditation practitioners before and after a 3-month retreat, Braboszcz, Cahn, Balakrishnan, Maturi, Grandchamp & Delorme (2013)	82	No	Digits	Letters	x	Stroop task; Global-local task	Wilcoxon ranksum test/regression
Improved control of exogenous attention in action video game	49	No	T1: White digit, T2: Black digit	Black letters	x	Anti-cuing task	GLMM

players, Cain, Prinzmetal, Shimamura & Landau (2014)							
How does bilingualism improve executive control? A comparison of active and reactive inhibition mechanisms, Colzato, Bajo, Van den Wildenberg, Paolieri, Nieuwenhuis, La Heij & Hommel (2008)	36	No	Digits	Letters	x	Stop-signal; Inhibition of return	RM-ANOVA (monolinguals vs. bilinguals)
Religion and the attentional blink: Depth of faith predicts depth of the blink, Colzato, Hommel & Shapiro (2010)	40	No	Digits	Letters	x	x	RM-ANOVA (atheists vs. Calvinists)
Dopamine and the management of attentional resources: Genetic markers of striatal D2 dopamine predict individual differences in the attentional blink, Colzato, Slagter, De Rover & Hommel (2011)	157	No	Digits	Letters	Genotyping	x	RM-ANOVA/regression
Blinks of the eye predict blinks of the mind, Colzato, Slagter, Spapé & Hommel (2008)	20	No	Digits	Letters	sEBR	x	RM-ANOVA (high vs. low sEBR))/correlation
Working memory and the attentional blink: Blink size is predicted by individual differences in operation span, Colzato, Spapé, Pannebakker & Hommel (2007)	80	No	Digits	Letters	x	OSPAN; Raven's SPM	RM-ANOVA/RM-ANCOVA/correlation/ regression
Individual differences in dispositional focus of attention predict attentional blink magnitude, Dale & Arnell (2010)	84	Yes	T1: White letter, T2: Black X	Letters	x	Global-local task	RM-ANOVA (high vs low local interference)/correlation
How reliable is the attentional blink? Examining the relationships within and between attentional blink tasks over time, Dale &	46	Yes; No	T1: Red letter, T2: Black X; Red letter	Black letters	x	x	RM-ANOVA/correlation

Arnell (2013)							
Multiple measures of dispositional global/local bias predict attentional blink magnitude, Dale & Arnell (2014)	39; 49****	Yes; No	T1: Red letter, T2: Black X; Red letter	Black letters	x	Various global-local tasks	RM-ANOVA (high vs low global score)/correlation/t-test
Individual differences within and across attentional blink tasks revisited, Dale, Dux & Arnell (2013)	118	Yes; No	Various	Various	x	x	RM-ANOVA/correlation/factor analysis
Distractor inhibition predicts individual differences in the attentional blink, Dux & Marois (2008)	48	No	T1: Red letter, T2: Green letter	White letters	x	x	RM-ANOVA/correlation/t-test
From sensory processes to conscious perception, Feinstein, Stein, Castillo & Paulus (2004)	16	Yes	T1: String of odd or even numbers, T2: Neutral or aversive word	Strings of letters	fMRI	x	RM-ANOVA/RM-ANCOVA (blinkers vs. non-blinkers)
The DRD2 C957T polymorphism and the attentional blink – A genetic association study, Felten, Montag, Kranczioch, Markett, Walter & Reuter (2013)	211	Yes	T1: Green letter, T2: Black X	Black digits	Genotyping	x	RM-ANCOVA
Developmental aspects of temporal and spatial visual attention: Insights from the attentional blink and visual search tasks, Garrad-Cole, Shapiro & Thierry (2010)	64; 16	No	Blue isosceles triangles pointing left/right or red isosceles triangles pointing up/down	Random colored shapes	x	Visual search task	RM-ANOVA (groups based on age)
Progressive age-related changes in the attentional blink paradigm, Georgiou-Karistianis, Tang, Vardy, Sheppard, Evans, Wilson, Gardner, Farrow & Bradshaw (2007)	50	No	Red letters	Black letters	x	National adult reading test; Beck depression inventory II	RM-ANOVA (groups based on age)/correlation
Action video game modifies visual selective attention, Green &	16; 17	Yes	T1: White letter, T2: Black X	Black letters	x	Flanker task; Enumeration task; Useful field of view task	RM-ANOVA (video game players vs. non video game

Bavelier (2003)							players)
Tracking the attentional blink profile: A cross-sectional study from childhood to adolescence, Heim, Benasich, Wirth & Keil (2013)	204	No	T1: Green sketches of transport means, T2: Green geometric shapes	White geometric figures and shapes	x	x	RM-ANOVA (groups based on grade)
Competition for cognitive resources during rapid serial processing: Changes across childhood, Heim, Wirth & Keil (2011)	45	No	Green symbols; letters	White symbols; letters	x	Raven's SPM; Digit span subtest (WISC III)	RM-ANOVA (young vs. old)/correlation
Effect of tobacco deprivation on the attentional blink in rapid serial visual presentation, Heinz, Waters, Taylor, Myers, Moolchan & Heishman (2007)	90	No	Red neutral words	Black neutral words	x	x	RM-ANOVA (smokers vs. deprived smokers vs. non-smokers)
Effect of cognitive aging on working memory consolidation, Jain & Kar (2014)	45	No	Letters	Digits; up/down arrow	x	MMSE; Rey's auditory verbal learning test; Logical memory test; Rey's complex figure test; Wisconsin card sorting test; Perceptual load task	RM-ANOVA (groups based on age)
No commonality between attentional capture and attentional blink, Kawahara & Kihara (2011)	135; 95	No	T1: Digit, T2: Digit; Digit outside central RSVP	Letters	x	Temporal and spatial visual search tasks	RM-ANOVA/correlation
Different attentional blink tasks reflect distinct information processing limitations: An individual differences approach, Kelly & Dux (2011)	39; 37	Yes; No	Various	Various	x	x	RM-ANOVA/t-test/correlation
Bilingualism and the increased attentional blink effect: Evidence that the difference between bilinguals and monolinguals generalizes to different levels of second language proficiency,	132	Yes	T1: White letter, T2: Black X	Black letters	x	LexTALE test of L2 proficiency; Raven's SPM; language background questionnaire	RM-ANOVA (high vs. low second language proficiency)/ correlation

Khare, Verma, Kar, Srinivasan & Brysbaert (2013)							
Individuals differ in the attentional blink: Mental speed and intra-subject stability matter, Klein, Arend, Beauducel & Shapiro (2011)	78; 58	No	T1: Red letter, T2: Blue letter	Black letters	x	Sternberg's memory search paradigm; N-back task; Predictive flexibility; Reactive flexibility; Pro- and anti-saccades; Psychometric intelligence; Span of apprehension task; Inspection time task; Visual search task	RM-ANOVA/correlation/ principcal component analysis
Simultaneous and preceding sounds enhance rapid visual targets: Evidence from the attentional blink, Kranczioch & Thorne (2013)	21	No	Letters	Meaningless shapes; masks: patterned squares	x	Cognitive failures questionnaire; NEO Fünf Faktoren Inventar; Test of attentional performance	RM-ANOVA/correlation
Age differences in the magnitude of the attentional blink, Lahar, Isaak & McArthur (2001)	55	Yes	T1: White target letter, T2: Red probe	Black target letters	x	Horn vocabulary test; Simple word span measures; Loaded word span measures	RM-ANOVA (young vs. old)
Attentional blink in adolescents with varying levels of impulsivity, Li, Chen, Lin & Yang (2005)	69	Yes	T1: Digit, T2: X-like character	Chinese characters	x	Barrat Impulsiveness Scale version 11; Maudsley obsessive compulsive inventory; Buss-perry aggression questionnaire; Children's depression inventory	RM-ANOVA (low vs. intermediate vs. high impulsivity)
Cognitive and attentional changes with age: evidence from attentional blink deficits, Maciokas & Crognale (2002)	32; 20	No	Letters	Digits	x	x	RM-ANOVA (young vs. old)
Personality predicts temporal attention costs in the attentional blink paradigm, MacLean & Arnell (2010)	29	No	T1: 5 repeated white uppercase or lowercase letters, T2: color word	Non-color neutral words	x	NEO personality inventory-revised; Emotion report form	t-test/regression/correlation
Individual differences in	67	Yes	T1: White letter,	Black letters	EEG	Time production task; Flanker	t-test/RM-ANOVA/correlation

electrophysiological responses to performance feedback predict AB magnitude, MacLean & Arnell (2013)			T2: Black X			task	
Dispositional affect predicts temporal attention costs in the attentional blink paradigm, MacLean, Arnell & Busseri (2010)	68	Yes	T1: Red letter; word; object picture, T2: Specified letter; word; object picture	Letters; words; object pictures	x	PANAS	RM-ANOVA/correlation/ regression
Resting EEG in alpha and beta bands predicts individual differences in attentional blink magnitude, MacLean, Arnell & Cote (2012)	30; 38	Yes	T1: 5 repeated white uppercase or lowercase letters; white letter, T2: color word; black X	Non-color neutral words; black letters	EEG	x	t-test/RM-ANOVA/correlation
Aging extents the time required to switch cognitive set, Male, Sheppard & Bradshaw (2009)	25	Yes	T1: Colored square, T2: Colored letter	Black letters	x	Kaufman brief intelligence test	RM-ANOVA/RM-ANCOVA (young vs. old)
A quick mind with letters can be a slow mind with natural scenes: Individual differences in attentional selection, Martens, Dun, Wyble, & Potter (2010)	29	No	Letters; Superordinate category pictures	Digits; Pictures of natural scenes	x	x	RM-ANOVA (blinkers vs. non-blinkers)
Cuing and stimulus probability effects on the P3 and the AB, Martens, Elmallah, London & Johnson (2006)	24; 17	No	T1: Frequent or infrequent letter; correctly cued or incorrectly cued letter, T2: Letter	Digits	EEG	x	RM-ANOVA/correlation
Working memory capacity, intelligence, and the magnitude of the attentional blink revisited, Martens & Johnson (2009)	97	No	Letters	Digits	x	Symmetry span test; Reading span test; Matrix span test; Letter span test; Raven's APM	RM-ANOVA/RM-ANCOVA/correlation/ regression
A quick visual mind can be a slow auditory mind: Individual differences in attentional selection	27	No	Letters; spoken letters	Digits; spoken digits	x	x	RM-ANOVA (blinkers vs. non-blinkers)

across modalities, Martens, Johnson, Bolle & Borst (2009)							
Restricted attentional capacity within but not between sensory modalities: An individual differences approach, Martens, Kandula & Duncan (2010)	56	No	Letters; spoken letters	Digits; spoken digits	x	x	RM-ANOVA/correlation
Quick minds slowed down: Effects of rotation and stimulus category on the attentional blink, Martens, Korucuoglu, Smid & Nieuwenstein (2010)	24; 24; 19	No	Letters (normal; rotated)	Digits (normal; rotated); letters (normal; rotated)	EEG	x	RM-ANOVA (blinkers vs. non-blinkers)
Quick minds don't blink: Electrophysiological correlates of individual differences in attentional selection, Martens, Munneke, Smid & Johnson (2006)	22; 22	No	Letters	Digits	EEG	x	RM-ANOVA (blinkers vs. non-blinkers); correlation
Individual differences in the attentional blink: The important role of irrelevant information, Martens & Valchev (2009)	28	No	Letters	Digits	x	Target-mask, target-mask task	RM-ANOVA (blinkers vs. non-blinkers)/t-test
Musical minds: Attentional blink reveals modality-specific restrictions, Martens, Wierda, Dun, De Vries & Smid (2015)	48	No	Spoken letters; letters	Spoken digits; digits	x	x	GLMM (musicians vs. non-musicians)/correlation
The attentional blink and P300, McArthur, Budd & Michie (1999)	14; 12	Yes	T1: White letter, T2: Black X	Black letters	EEG	x	RM-ANOVA/correlation
The attentional blink is immune to masking-induced data limits, McLaughlin, Shore & Klein (2001)	16	No	Letters	Digits	x	Target-mask, target-mask task	RM-ANOVA/correlation
The attentional blink in developing readers, McLean, Stuart, Visser & Castles (2009)	86	No	1 out of 5 shapes	Keyboard symbols & random dot patches	x	Three reading measures; Non-verbal IQ; RAN	RM-ANOVA/correlation/ regression

Eliminating the attentional blink through binaural beats: A case for tailored cognitive enhancement, Reedijk, Bolders, Colzato & Hommel (2015)	24	No	Digits	Letters	sEBR	x	RM-ANOVA (low vs. high EBR)
Resource sharing in the attentional blink, Shapiro, Schmitz, Martens, Hommel & Schnitzler (2006)	10	No	X/O; L/T	White letters	MEG	x	RM-ANOVA/correlation
Using the attention cascade model to probe cognitive aging, Shih (2009)	42	No	Digits	Letters	x	x	RM-ANOVA (young vs. old)
Distractor inhibition predicts individual differences in recovery from the attentional blink, Slagter & Georgopoulou (2013)	40	No	T1: Red letter, T2: Green letter	White uppercase letters	sEBR	x	RM-ANOVA/correlation
Neural competition for conscious representation across time: An fMRI study, Slagter, Johnstone, Beets & Davidson (2010)	16; 24	No	T1: Body without a head, T2: Natural scene	Scrambled scene images	fMRI	x	RM-ANOVA/t-test
Mental training affects distribution of limited brain resources, Slagter, Lutz, Greischar, Francis, Nieuwenhuis, Davis, Davidson (2007)	40	No	Digits	Letters	EEG	x	RM-ANOVA/RM-ANCOVA (novices vs. practitioners)/ correlation
Theta phase synchrony and conscious target perception: Impact of intensive mental training, Slagter, Lutz, Greischar, Nieuwenhuis & Davidson (2009)	40	No	Digits	Letters	EEG	x	RM-ANOVA (novices vs. practitioners)/Wilcoxon sign rank test
PET evidence for a role for striatal dopamine in the attentional blink: Functional implications, Slagter, Tomer, Christian, Fox, Colzato, King, Murali & Davidson (2012)	14	No	Digits	Letters	PET	x	RM-ANOVA/correlation
The more your mind wanders, the	121;	No	Letters	Digits	x	Sustained attention to	RM-ANOVA/correlation

smaller your attentional blink: An individual differences study, Thomson, Ralph, Besner & Smilek (2014)	102					response task; Mind wandering spontaneous and deliberate questionnaires	
Evidence for mental ability related individual differences in the attentional blink obtained by an analysis of the P300 component, Troche, Indermühle & Rammsayer (2012)	60	Yes	T1: Yellow letter, T2: Digit “2”	White letters	EEG	Berlin intelligence structure test	RM-ANOVA (high vs. low mental ability)
Attentional blink and impulsiveness: Evidence for higher functional impulsivity in non-blinkers compared to blinkers, Troche & Rammsayer (2013)	30	Yes	T1: Yellow letter, T2: Digit “2”	White letters	EEG	Dickman’s impulsivity inventory	RM-ANOVA/t-test (blinkers vs. non-blinkers)
Age effects on attentional blink performance in meditation, Van Leeuwen, Müller & Melloni (2009)	51	Yes	T1: Red digit, T2: Black digit	Letters	x	x	RM-ANOVA (groups based on age and meditation experience)
Control over experience? Magnitude of the attentional blink depends on meditative state, Van Vugt & Slagter (2014)	30	No	Digits	Letters	x	Five factor mindfulness Questionnaire; questionnaire meditation experience; Beck Depression Inventory; PANAS; State-trait anxiety inventory-trait	RM-ANOVA (FA meditation vs. OM meditation)
How does information processing speed relate to the attentional blink?, Visser & Ohan (2012)	69	No	Letters	Random dot patches & digits & keyboard symbols	x	Rapid letter naming test (RAN)	RM-ANOVA (low RAN score vs. high RAN score)/correlation
Relations between the attentional blink and aspects of psychometric intelligence: A fixed-links modeling approach, Wagner, Rammsayer, Schweizer & Troche (2014)	201	Yes	T1: Yellow letter, T2: Digit “2”	White letters	x	Berlin intelligence structure test	RM-ANOVA/fixed-links modeling
A fixed-links modeling approach	201	Yes	T1: Yellow letter,	White	EEG	x	RM-ANOVA/ fixed-links

to assess individual differences in the attentional blink: Analysis of behavioral and psychophysiological data, Wagner, Rammsayer, Schweizer & Troche (2015)			T2: Digit “2”	letters			modeling
Individual differences in the attentional blink: The temporal profile of blinkers and non-blinkers, Willems, Wierda, Van Viegen & Martens (2013)	28; 132	No	Red letters	Black letters	x	x	RM-ANOVA/RM-ANCOVA (Experiment 1: blinkers vs. non-blinkers)
Aerobic fitness and the attentional blink in preadolescent children, Wu & Hillman (2013)	39	No	White digits	White letters	EEG	Kaufman brief intelligence test; Eidenburgh handedness inventory; Cardiorespiratory fitness assessment	RM-ANOVA (high-fit vs. low-fit children)

* The reported N is the sample size after exclusion of any participants

** The extra tasks were either tested within the same sample of participants as tested in the AB task, or in a (partially) different sample

*** Only the main statistical methods that are used per study are mentioned, so for example post-hoc tests are omitted. If the sample was somehow grouped, this is indicated within parentheses.

Note: RM-ANOVA – Repeated Measures Analysis of Variance; RM-ANCOVA – Repeated Measures Analysis of Covariance - GLMM – Generalized Linear Mixed Model

**** Multiple numbers divided by “;” refers to multiple experiments
